# A model-guided symbolic execution approach for network protocol implementations and vulnerability detection

**DOI:** 10.1371/journal.pone.0188229

**Published:** 2017-11-16

**Authors:** Shameng Wen, Qingkun Meng, Chao Feng, Chaojing Tang

**Affiliations:** College of Electronic Science and Engineering, National University of Defense Technology, Changsha, China; Tianjin University, CHINA

## Abstract

Formal techniques have been devoted to analyzing whether network protocol specifications violate security policies; however, these methods cannot detect vulnerabilities in the implementations of the network protocols themselves. Symbolic execution can be used to analyze the paths of the network protocol implementations, but for stateful network protocols, it is difficult to reach the deep states of the protocol. This paper proposes a novel model-guided approach to detect vulnerabilities in network protocol implementations. Our method first abstracts a finite state machine (FSM) model, then utilizes the model to guide the symbolic execution. This approach achieves high coverage of both the code and the protocol states. The proposed method is implemented and applied to test numerous real-world network protocol implementations. The experimental results indicate that the proposed method is more effective than traditional fuzzing methods such as SPIKE at detecting vulnerabilities in the deep states of network protocol implementations.

## Introduction

Network protocol implementations are often prone to vulnerabilities, and formal verification techniques cannot address the problems in the implementations. Fuzz testing and symbolic execution are widely applied to detect vulnerabilities in network protocol implementations. However, it is difficult to reach the deep states of the stateful network protocols given the complex interactions and state transitions of these methods, because they do not fully exploit the packet interaction and state transition information.

**Fig 1:**
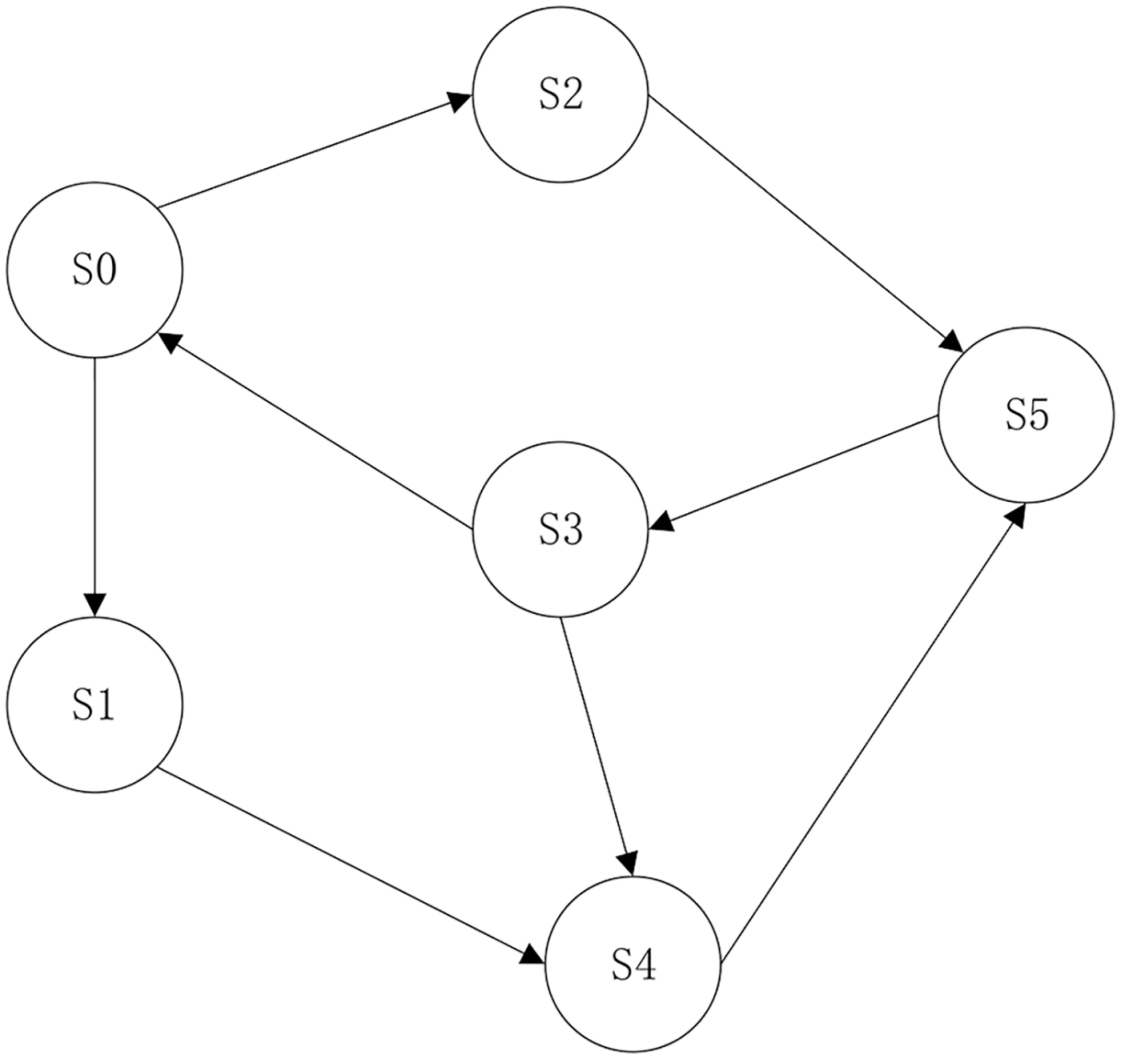
FSM of a stateful protocol.

In this article, we propose a novel approach that uses an FSM model to guide the symbolic execution. More precisely, we utilize the L* [1] online learning algorithm to construct the FSM model. The FSM for a stateful protocol is presented in Fig 1.

We first build a prototype model-guided symbolic execution system to explore the protocol states and detect vulnerabilities in the deep states of the protocol. Then, we use the prototype system to test several real-world network protocol implementations and compare this system with the traditional fuzzing tool SPIKE.

## Related work

In this section, we briefly present research works related to the current study [2, 3]. Fuzzing has been used to detect network protocol implementation vulnerabilities for more than 20 years [4]. Several tools exist that are specifically aimed at network protocol implementations. SPIKE [5] developed by Dave Aitel, is a framework that provides an API to assist in creating fuzzed network protocol implementations. PROTOS [6], developed by the Oulu University Secure Programming Group, generates input packets intelligently based on protocol specifications. However, these methods do not model stateful protocols and cannot reach the deep states of network protocols.

Symbolic execution is a powerful technique for analyzing program behavior, identifying bugs, and generating tests [7]. The main concept underlying symbolic execution is to use symbolic input values instead of concrete input values. This approach treats the paths as symbolic constraints and solves the constraints to produce a concrete input as a test case. Symbolic execution has been applied to test network protocol implementations. SymNV [8] and SymbexNet [9], developed by JaeSeung Song, combine symbolic execution with rule-based specifications based on KLEE [10], a symbolic engine tool. KleeNet [11] integrates KLEE to detect vulnerabilities in wireless sensor networks. SymNet [12], proposed by Raimonds Sasnauskas, is a testing environment for unmodified protocol implementations running on diverse operating systems. It was designed on top of the S2E platform [13], runs on the QEMU [14] virtual machine, and adopts KLEE as its symbolic engine.

Traditional fuzzing and symbolic execution methods do not make full use of the protocol state information; thus, they have difficulty reaching the deep states of network protocol implementations. Jingling Zhao [15] proposed a smart fuzzing algorithm based on a regression finite state machine (RFSM-Fuzzing) that can test the robustness of wireless network protocols and find potential flaws. MACE [16] uses concolic execution [17] to build an abstract FSM model to guide further state exploration. Our previous work [18] that combines network analysis and binary reverse engineering is an advanced fuzzing testing method for detecting vulnerabilities in network protocols. However, without making full use of the protocol state information, this method cannot reach the deep states of the network protocol. As a result, the vulnerabilities related to the deep states cannot be detected by this method.

## Background

### Protocol inference

A state machine model is the standard way to model a protocol. To infer the protocol, state machine inferences are used to extract a state machine model for each network protocol implementation. This approach learns the state machine by sending network protocol packets and observing the response packets. There are two types of FSMs. The first type is called the Moore machine [19] and the second type is called the Mealy machine [20]. The Moore machine’s outputs depend solely on the current state, while the Mealy machine’s outputs depend on both the current state and on the inputs. Because protocol states use the input and output packets to interact with their environment, the Mealy machine is more suitable for making protocol inferences.

**Inference algorithm**: L* was the first learning automata algorithm to use an active approach [1], and it is still the most widely-used active approach for performing formal verifications. Niese proposed a modified version of the L* algorithm to infer Mealy machines [21] that involves a teacher and a learner. The teacher possesses knowledge about a deterministic Mealy machine, while the learner has no knowledge about the Mealy machine except for its input and output. The learner learns about the Mealy machine by querying the teacher.

### Symbolic execution

James proposed symbolic execution in 1976 [22], which takes input in the form of symbolic values rather than concrete values. The path constraints utilize the conditions of each path, which depend upon the symbolic values. They are collected as symbolic value expressions from the starting point to the current point. The constraints are resolved by the constraint solver, leading to a concrete value as an input. The concrete input enables program execution to the current point.

**Fig 2:**
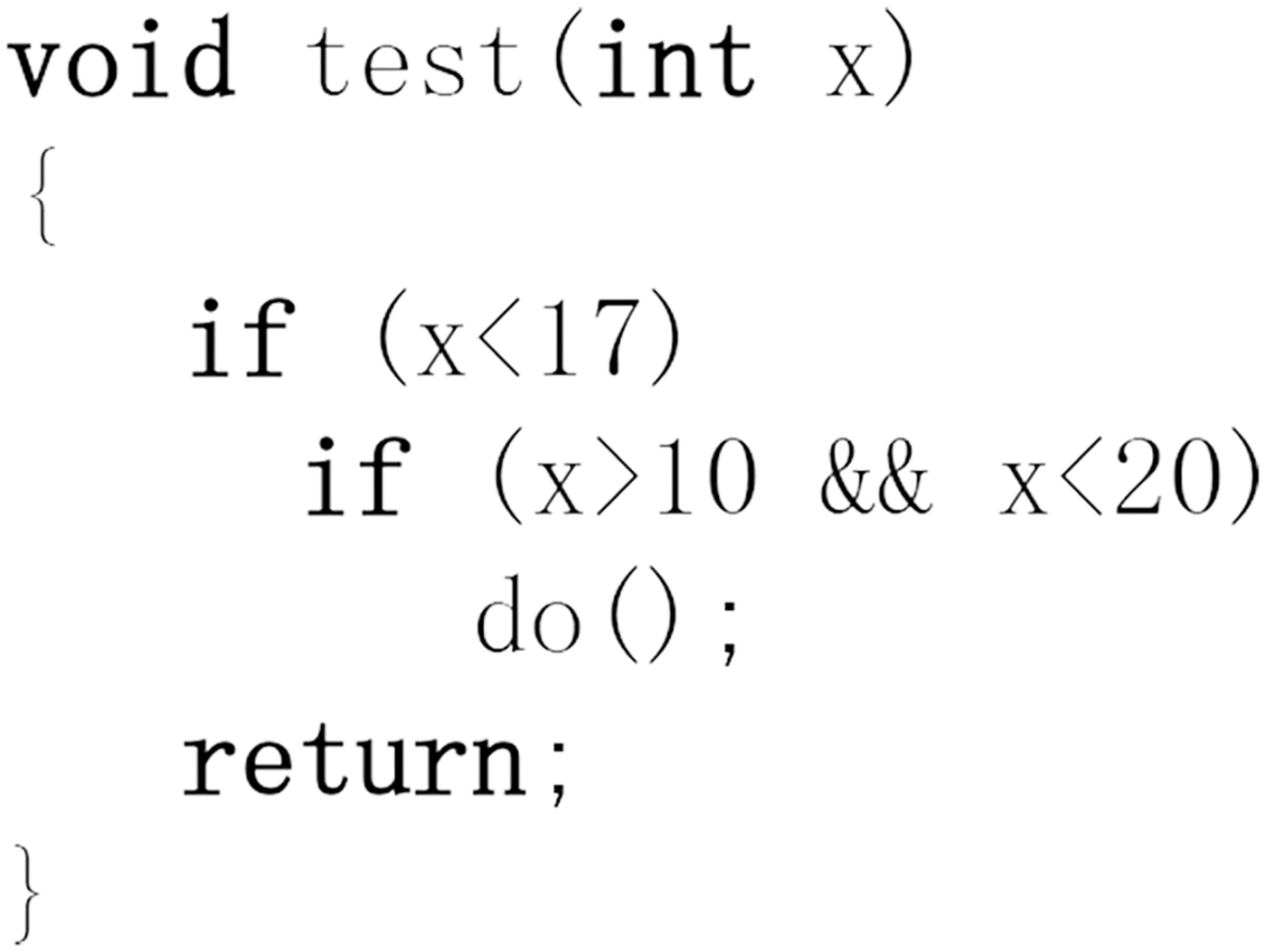
An example of symbolic execution example code.

**Fig 3:**
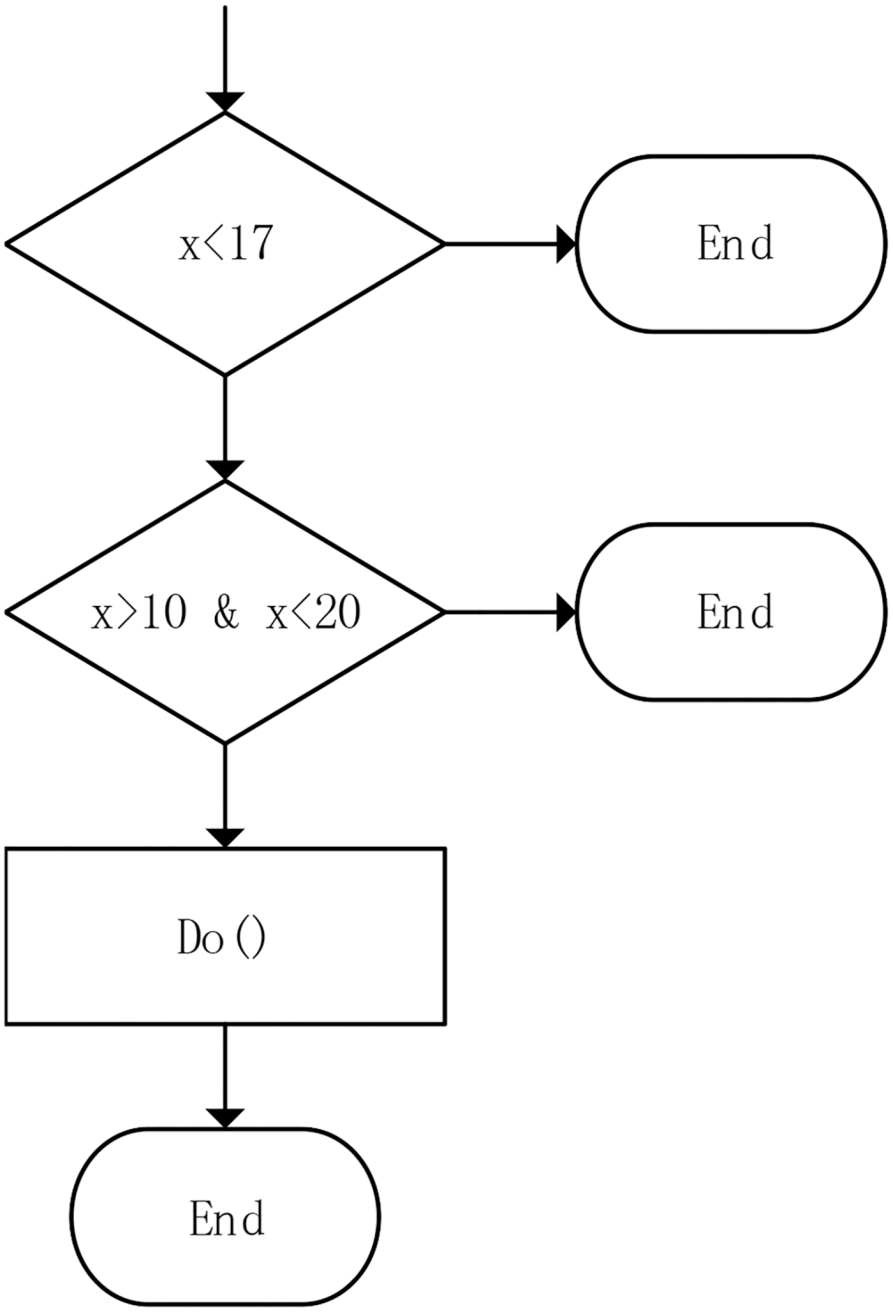
An example of symbolic execution example flow diagram.

Symbolic execution is an enhanced testing technique because it is more efficient than normal testing. It requires testing each path only once due to the symbolic value, which achieves the same effect as testing a path using all the concrete values that match the path constraints. An example of symbolic execution is shown in Figs 2 and 3: when symbolic execution results in a 5, the symbolic expression is (*x* < 17) ∧ (*x* > 10 ∧ *x* < 20)—the same as when the path is executed with the concrete values *x* = 11, 12, 13, 14, 15, 16.

## Framework design

This study presents a new model-guided symbolic execution approach to detect flaws in network protocol implementations. The basic concept is to link the program paths and states of the protocol using an FSM to guide the symbolic execution. This approach helps in exploring the deep states of network protocol implementations.

**Fig 4:**
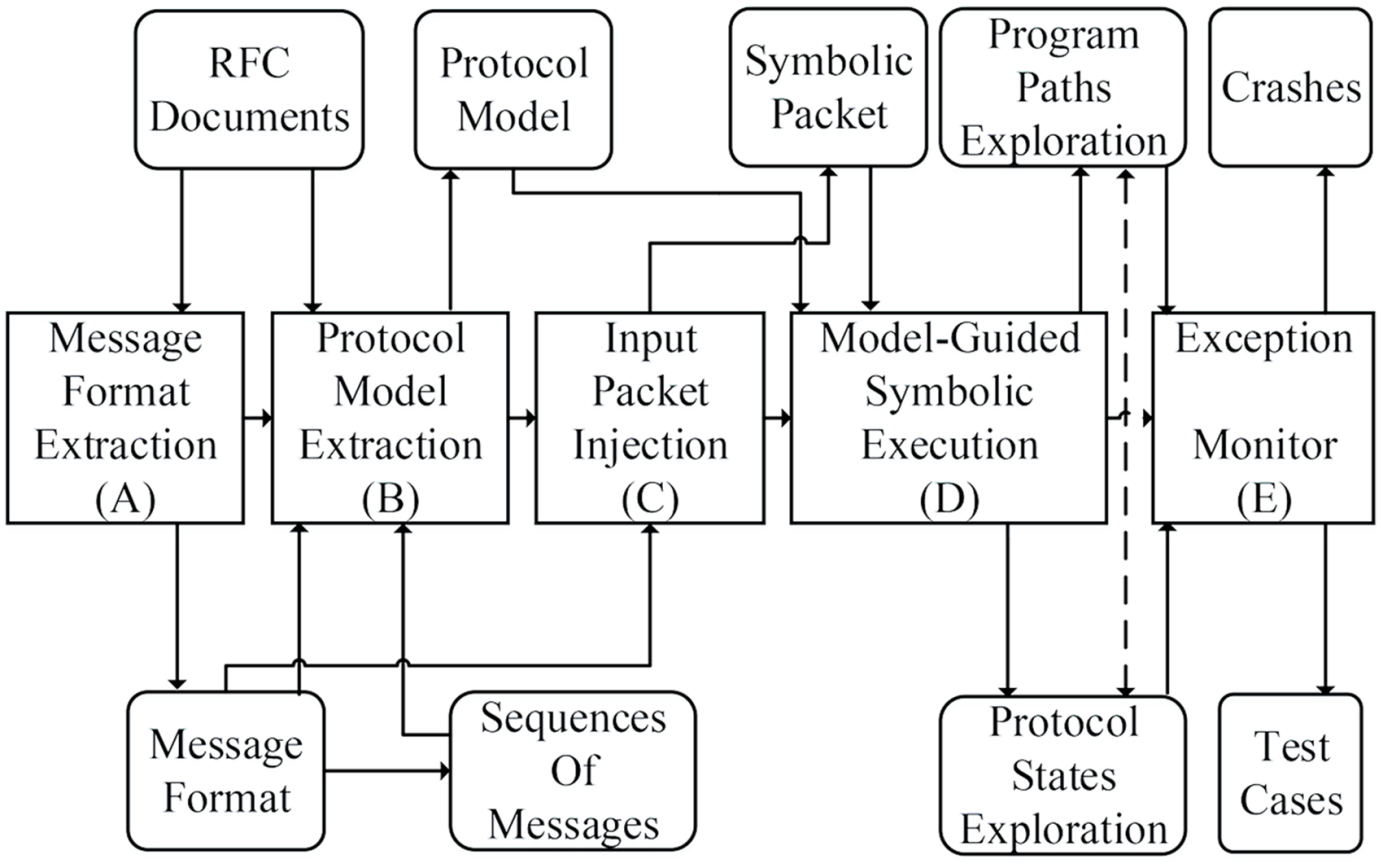
Framework of proposed method.

Fig 4 presents the framework of our method, which consists of the following components: a message format extractor, a protocol model extractor, an input packet injector, a model-guided symbolic executor, and an exception monitor.

The method proceeds as follows: first, we extract the message formats from the protocol specification of the target network protocol implementation. Second, we automatically infer an abstract FSM of the network protocol implementation. Third, after acquiring the message formats and the protocol model, we use the message formats to construct symbolic packets, which are used as the input of symbolic execution, and we leverage the protocol model to guide the symbolic execution to improve the coverage of both program paths and protocol states. Finally, we monitor the symbolic execution procedure; when exceptions occur, we report the crashes and record the test cases.

### Message format extractor

**Fig 5:**
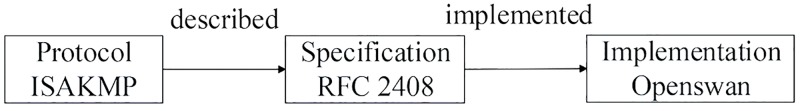
The relationship among protocol, specification and implementation of ISAKMP protocol.

**Fig 6:**
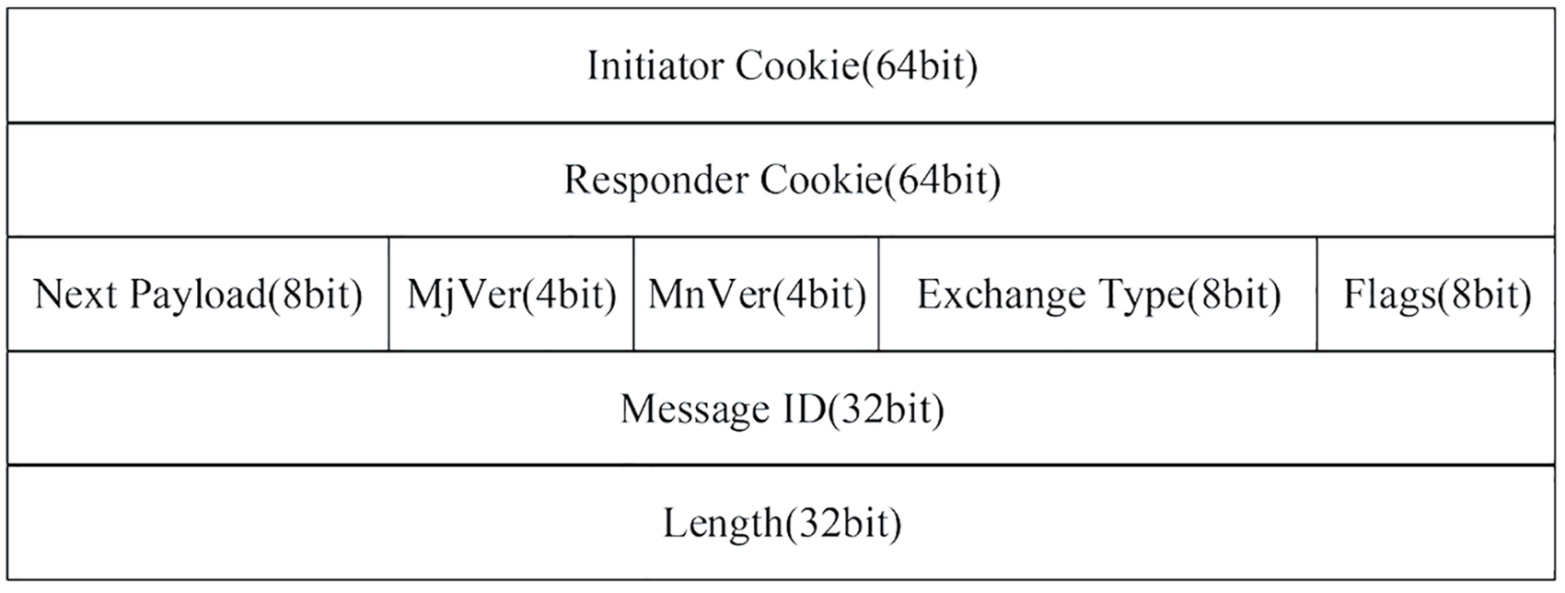
The message format of ISAKMP header.

A Request for Comments (RFC) is a document that provides a description of a protocol specification. The document contains descriptions of the services, types and formats of messages exchanged and the rules governing the reactions of the entities involved. Network developers have implemented many different network protocol software applications based on these standard protocol specifications. The relationship between the protocol description, its specification, and the implementation of the ISAKMP protocol is shown in Fig 5. This protocol is described in RFC2408 [23] and has been implemented as Openswan, Wireshark, Adaptive Security Appliances, and OpenBSD. The packet format of an ISAKMP header is depicted in Fig 6.

We analyze the RFC documents of the target network protocol implementations by focusing on the description of the message formats. In our work, the message formats are manually extracted from the RFC documents.

### Protocol model extractor

**Fig 7:**
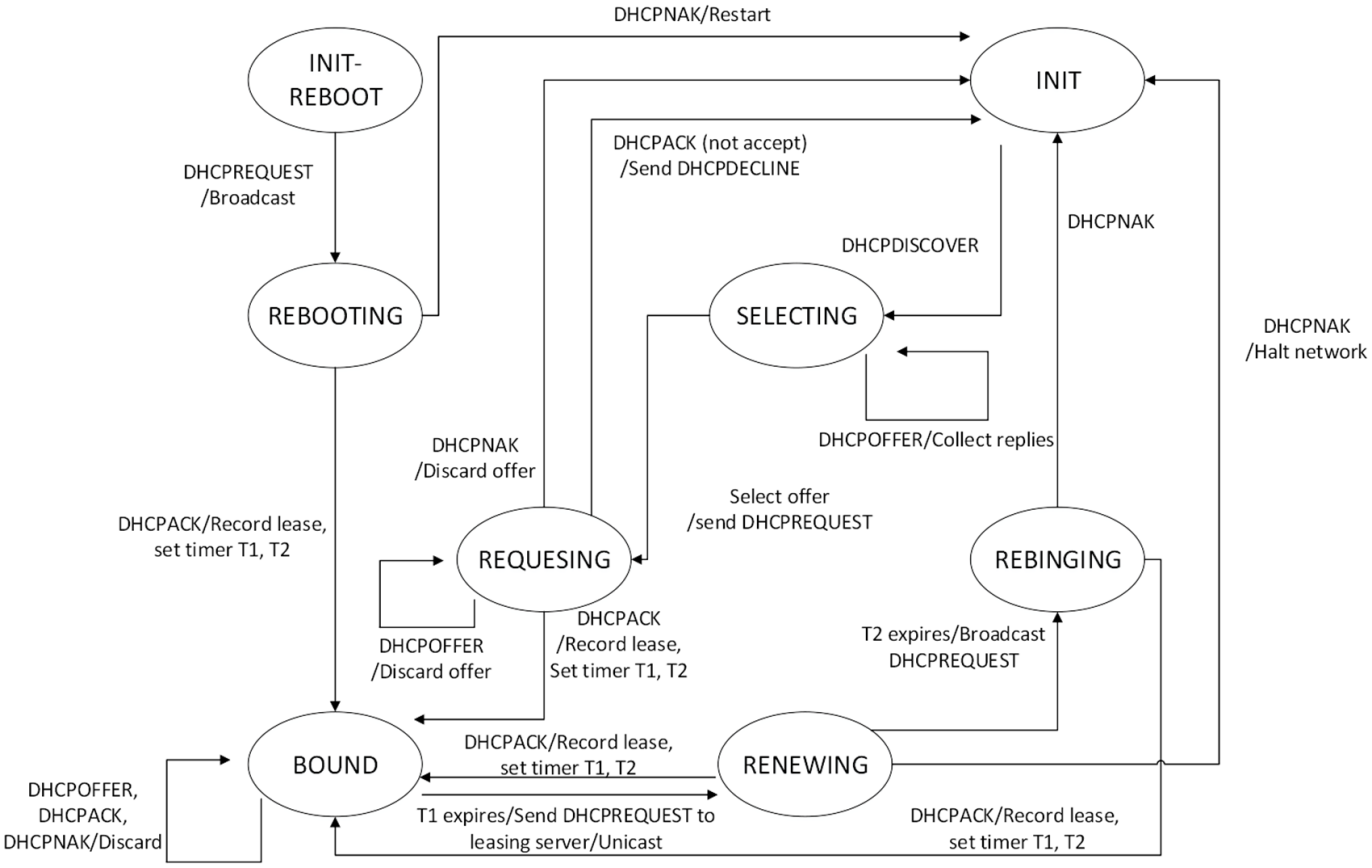
FSM of DHCP protocol.

We leverage the L* inference algorithm to infer the FSM model for the network protocol implementation. L* probes a black box with a sequence of messages, listens to the responses, and builds an FSM from the responses [16]. The component called the protocol model extractor learns an abstract model of the target implementation of the network protocol by producing a sequence of messages used as input. The produced input is further modified based on the network protocol implementation, and the output description. The proposed model utilizes the message format extracted by the message format extractor component to generate many input messages as the starting inputs to infer the first FSM. Subsequently, the FSM guides the symbolic execution to explore the possible states of the network protocol implementation. The symbolic execution produces numerous input as well as output messages. These messages are employed to further infer and evolve the FSM, and this is a repetitive process. If the target is a known protocol, we can acquire the RFC documents and use them to assist the FSM in inferring the model. The proposed method produces a more accurate the model. The FSM of the DHCP protocol is illustrated in Fig 7.

### Input packet injector

To leverage symbolic execution for network protocol implementations, we need to inject the input packet into memory by intercepting the network-related functions and their arguments, as shown in Table 1. In this method, we can inject the input packet into memory without changing or recompiling the source code of the network protocol implementations.

**Table 1:** Network-related functions.

send()	recv()	socket()	accept()
sendmsg()	read()	sendto()	poll()
recvmsg()	write()	select()	recvfrom()
close()	connect()	listen()	bind()

**Fig 8:**
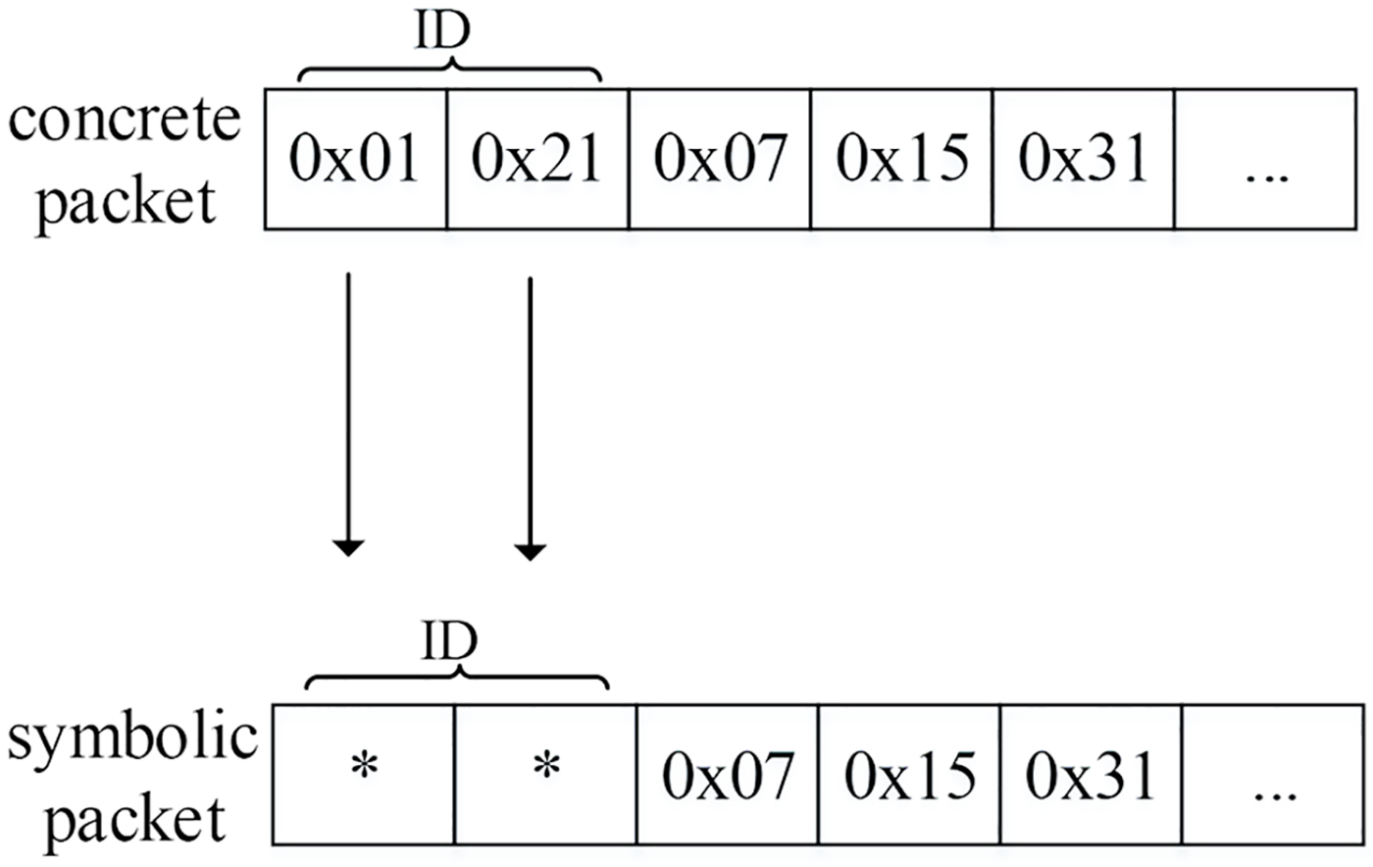
An example of packet marking.

After injecting the input packet into memory, the packets are marked as symbolic packets by replacing some of the bytes of concrete input packets with symbolic values. To reduce the path explosion problem, specific interesting bytes can be selected for replacement. Fig 8 depicts an example of packet marking in which the ID field is targeted for symbolic values.

### Model-guided symbolic executor

**Fig 9:**
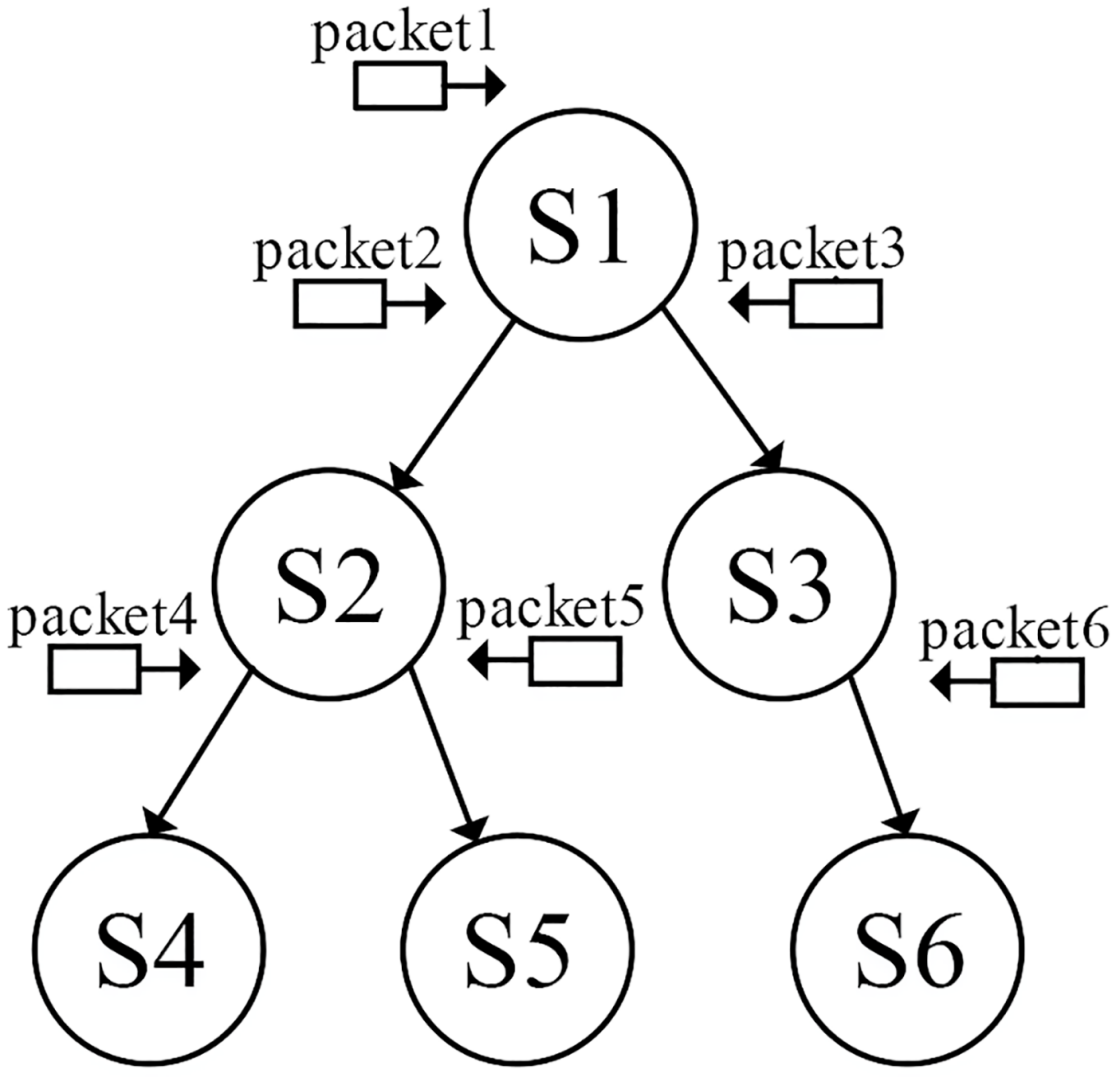
The relationship between protocol states and program paths protocol states.

**Fig 10:**
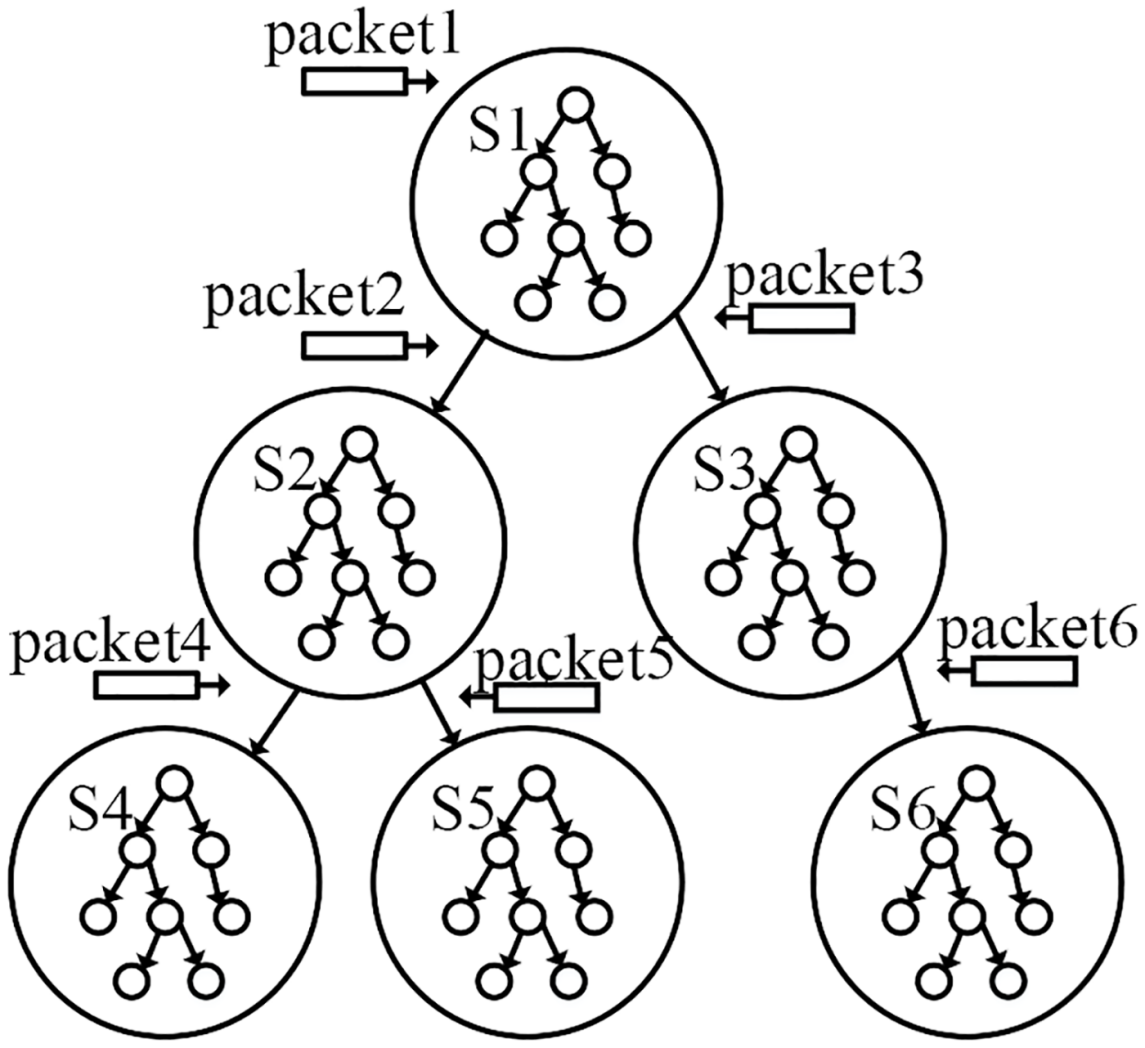
The relationship between protocol states and program paths program paths.

The model-guided symbolic execution leverages the FSM to guide the symbolic execution to concentrate on exploring the interesting states. With FSM, we can easily obtain an input packet sequence to reach any state of the FSM to build the relationships between program paths and protocol states. This link is shown in Figs 9 and 10, where the program states are depicted in Figs 9 and 10 represents the program paths. From the protocol model extraction component we acquire the FSM of the network protocol implementation. Then, the model-guided symbolic execution module leverages the FSM model to guide the symbolic execution. As shown in Figs 9 and 10, the conventional symbolic execution approach can become stuck in the program paths of state S1; however, the module-guided symbolic execution leverages the FSM to inject packet 2, causing the program to transition to state S2. Similarly, if we intend to explore state S4, we can inject packet 4.

### Exception monitor

The exception monitor component tracks the test cases and produces a crash report. This module leverages dynamic kernel instrumentation to catch unhandled exceptions. It also tracks CPU states and the execution context, which are useful for analysis in the later stages. Default exception handling functions can be called for unhandled exceptions by the network protocol implementations. In the proposed work, these functions are used to track test cases and produce the crash report. For WINDOWS system, we intercept the default exception handler function: UnhandledExceptionFilter. For *nix system, we intercept the exit function of the process, whose address can be acquired from System.map file.

## Implementation and evaluation

### Implementation

The proposed method is implemented based on S2E, and it is a full-system selective symbolic execution platform [13]. S2E enables the reuse of parts of the QEMU virtual machine [14], the KLEE symbolic execution engine [10], the LLVM tool chain [24], and the STP constraint solver [25].

This paper proposes an extension to S2E to leverage model-guided symbolic execution and find flaws in network protocol implementations. To infer the state machines for the network protocol implementations, we adopt LearnLib [26], which uses a modified version of the L* algorithm.

### Evaluation

We evaluate the effectiveness of our proposed method by testing several known vulnerabilities in real-world File Transfer Protocol (FTP) implementations(S1 File). We compare our proposed method with the general fuzzing tool SPIKE(S2 File).

The experiments were conducted by hosting the QEMU virtual platform on two machines, namely, a host machine and a guest machine. The host machine includes an Intel Core i7-7700K 4.2 GHz CPU with 32 GB memory and was running Ubuntu 14.04. The guest machine was running Debian Linux 7 and Windows XP SP3. Our prototype system and the traditional fuzzing tool SPIKE were running on the host machine.

Comparisons of the results of the prototype system and SPIKE for vulnerability detection are shown in Table 2. The empirical results indicate that our prototype system can detect the known vulnerabilities and more effectively than SPIKE.

**Table 2:** Comparison of the prototype system and SPIKE in vulnerability detection on FTP implementations.

Software Name	Software Version	Vulnerability Number	Vulnerability Type	The Prototype System	SPIKE
Core FTP	2.2 build 1798	CVE-2014-4643	Integer Overflow	Yes	Yes
TYPSoft FTP Server	1.1	CVE-2012-5329	Buffer Overflow	No	No
AmmSoft ScriptFTP	3.3	CVE-2011-3976	Buffer Overflow	Yes	No
Labtam ProFTP	2.9	CVE-2009-3976	Buffer Overflow	Yes	Yes
Detection Rate				75%	50%

## Conclusion

This study presented a new method based on model-guided symbolic execution. The proposed method associates program paths with protocol states. It uses an FSM to guide the symbolic execution, allowing it to analyze interesting deep states of network protocol implementations. We built a prototype system and used it to test for several known vulnerabilities that exist in real-world implementations of various network protocols. The empirical results indicate that the proposed method is effective at detecting vulnerabilities.

In possible future work, we plan to use our method to test several other network protocols and to seek to improve the efficiency of our method.

## Supporting information

S1 File FTP programs. (ZIP)

S2 File SPIKE. (ZIP)

## References

[1] Angluin D. Learning regular sets from queries and counterexamples. Information and computation. 1987;75(2):87–106. doi: 10.1016/0890-5401(87)90052-6

[2] Tang S, Lee BS, He B. Dynamicmr: A dynamic slot allocation optimization framework for mapreduce clusters. IEEE Transactions on Cloud Computing. 2014;2(3):333–347. doi: 10.1109/TCC.2014.2329299

[3] Tang S, Yu C, Sun J, Lee BS, Zhang T, Xu Z, et al Easypdp: An efficient parallel dynamic programming runtime system for computational biology. IEEE Transactions on Parallel and Distributed Systems. 2012;23(5):862–872. doi: 10.1109/TPDS.2011.218

[4] Barton JH, Czeck EW, Segall ZZ, Siewiorek DP. Fault injection experiments using FIAT. IEEE Transactions on Computers. 1990;39(4):575–582. doi: 10.1109/12.54853

[5] Aitel D. The advantages of block-based protocol analysis for security testing. Immunity Inc, February. 2002;105:106.

[6] Kaksonen R, Laakso M, Takanen A. Software security assessment through specification mutations and fault injection In: Communications and Multimedia Security Issues of the New Century. Springer; 2001 p. 173–183. Available from: http://link.springer.com/chapter/10.1007/978-0-387-35413-2_16.

[7] Chipounov V, Georgescu V, Zamfir C, Candea G. Selective symbolic execution. In: Proceedings of the 5th Workshop on Hot Topics in System Dependability (HotDep); 2009. Available from: https://infoscience.epfl.ch/record/139393.

[8] Song J, Ma T, Cadar C, Pietzuch P. Rule-based verification of network protocol implementations using symbolic execution. In: Computer Communications and Networks (ICCCN), 2011 Proceedings of 20th International Conference on. IEEE; 2011. p. 1–8. Available from: http://ieeexplore.ieee.org/abstract/document/6005945/.

[9] Song J, Cadar C, Pietzuch P. SYMBEXNET: testing network protocol implementations with symbolic execution and rule-based specifications. IEEE Transactions on Software Engineering. 2014;40(7):695–709. doi: 10.1109/TSE.2014.2323977

[10] Cadar C, Dunbar D, Engler DR, others. KLEE: Unassisted and Automatic Generation of High-Coverage Tests for Complex Systems Programs. In: OSDI. vol. 8; 2008 p. 209–224. Available from: http://static.usenix.org/legacy/events/osdi08/tech/full_papers/cadar/cadar_html/paper.html.

[11] Sasnauskas R, Landsiedel O, Alizai MH, Weise C, Kowalewski S, Wehrle K. KleeNet: discovering insidious interaction bugs in wireless sensor networks before deployment. In: Proceedings of the 9th ACM/IEEE International Conference on Information Processing in Sensor Networks. ACM; 2010. p. 186–196. Available from: http://dl.acm.org/citation.cfm?id=1791235.

[12] Sasnauskas R, Kaiser P, Jukić RL, Wehrle K. Integration testing of protocol implementations using symbolic distributed execution. In: Network Protocols (ICNP), 2012 20th IEEE International Conference on. IEEE; 2012. p. 1–6. Available from: http://ieeexplore.ieee.org/abstract/document/6459940/.

[13] Chipounov V, Kuznetsov V, Candea G. S2E: A platform for in-vivo multi-path analysis of software systems. ACM SIGPLAN Notices. 2011;46(3):265–278.

[14] Bellard F. QEMU, a fast and portable dynamic translator. In: USENIX Annual Technical Conference, FREENIX Track; 2005. p. 41–46. Available from: https://www.usenix.org/legacy/event/usenix05/tech/freenix/full_papers/bellard/bellard_html/.

[15] Zhao J, Chen S, Liang S, Cui B, Song X. RFSM-Fuzzing a Smart Fuzzing Algorithm Based on Regression FSM. In: P2P, Parallel, Grid, Cloud and Internet Computing (3PGCIC), 2013 Eighth International Conference on. IEEE; 2013. p. 380–386. Available from: http://ieeexplore.ieee.org/abstract/document/6681258/.

[16] Cho CY, Babic D, Poosankam P, Chen KZ, Wu EX, Song D. MACE: Model-inference-Assisted Concolic Exploration for Protocol and Vulnerability Discovery. In: USENIX Security Symposium; 2011. p. 139–154. Available from: https://www.usenix.org/event/sec11/tech/full_papers/Cho.pdf.

[17] Sen K, Marinov D, Agha G. CUTE: a concolic unit testing engine for C In: ACM SIGSOFT Software Engineering Notes. vol. 30 ACM; 2005 p. 263–272. Available from: http://dl.acm.org/citation.cfm?id=1081750.

[18] Fernandes JF, Goubergrits L, Brüning J, Hellmeier F, Nordmeyer S, da Silva TF, et al Beyond Pressure Gradients: The Effects of Intervention on Heart Power in Aortic Coarctation. PloS one. 2017;12(1):e0168487 doi: 10.1371/journal.pone.0168487 28081162 10.1371/journal.pone.0168487 PMC5231370

[19] Moore EF. Gedanken-experiments on sequential machines. Automata studies. 1956;34:129–153.

[20] Mealy GH. A method for synthesizing sequential circuits. Bell Labs Technical Journal. 1955;34(5):1045–1079. doi: 10.1002/j.1538-7305.1955.tb03788.x

[21] Niese O. An integrated approach to testing complex systems. Technical University of Dortmund, Germany; 2003 Available from: https://eldorado.tu-dortmund.de/bitstream/2003/2545/2/Niese.pdf.

[22] King JC. Symbolic execution and program testing. Communications of the ACM. 1976;19(7):385–394. doi: 10.1145/360248.360252

[23] Maughan D, Schneider M. Internet security association and key management protocol (ISAKMP). 1998;.

[24] Lattner C, Adve V. LLVM: A compilation framework for lifelong program analysis & transformation. In: Proceedings of the international symposium on Code generation and optimization: feedback-directed and runtime optimization. IEEE Computer Society; 2004. p. 75. Available from: http://dl.acm.org/citation.cfm?id=977673.

[25] Ganesh V, Dill DL. A decision procedure for bit-vectors and arrays In: CAV. vol. 4590 Springer; 2007 p. 519–531. Available from: http://link.springer.com/content/pdf/10.1007/978-3-540-73368-3.pdf#page=533.

[26] Raffelt H, Steffen B, Berg T, Margaria T. LearnLib: a framework for extrapolating behavioral models. International Journal on Software Tools for Technology Transfer (STTT). 2009;11(5):393–407. doi: 10.1007/s10009-009-0111-8

